# An Exploratory Study on Imaging Resolution, Operational Parameters, and Measurement Uncertainty in UAV-Based Crack Inspection

**DOI:** 10.3390/s26031031

**Published:** 2026-02-05

**Authors:** Suk Bae Lee, Dong Ha Lee, Jisung Kim

**Affiliations:** 1 Department of Civil & Infrastructure Engineering, College of Construction and Environmental Engineering, Gyeongsang National University, Jinju 52828, Republic of Korea; sukbaelee@gnu.ac.kr; 2 Department of Integrated Energy and Infra System, Kangwon National University, Chuncheon 24341, Republic of Korea; geodesy@kangwon.ac.kr; 3 School of Geography, University of Leeds, Leeds LS2 9JT, UK

**Keywords:** UAV inspection, crack detection, GSD, flight efficiency

## Abstract

Unmanned aerial vehicles (UAVs) are increasingly used for crack inspection of civil infrastructure. However, crack interpretation from UAV imagery is constrained by trade-offs among imaging resolution, operational efficiency, and measurement uncertainty. Higher resolution generally requires reduced flight distance, increased image quantity, and greater data-processing effort, which can limit inspection efficiency. This study presents an exploratory analysis of UAV-based crack inspection from a measurement-oriented perspective. Empirical UAV flight experiments were conducted to examine the relationships among flight distance, ground sampling distance (GSD), image quantity, and photogrammetric processing effort under controlled acquisition conditions. In addition, a dataset-based segmentation analysis was performed to investigate pixel-level uncertainty associated with crack thickness representation near the resolution limit. This analysis does not aim to estimate physical crack width, but rather to identify intrinsic limitations of image-based crack interpretation. The results indicate that while flight distance and GSD follow expected geometric relationships, image quantity and processing effort are influenced by multiple interacting factors rather than resolution alone. Pixel-level analysis further reveals substantial segmentation uncertainty for thin cracks represented by only a few pixels. These findings highlight the importance of accounting for measurement uncertainty and operational trade-offs when planning efficient UAV-based crack inspections.

## 1. Introduction

Unmanned aerial vehicles (UAVs) have been increasingly adopted for safety inspection of civil infrastructure due to their flexibility and ability to access large or hard-to-reach structures. Among various inspection tasks, crack inspection remains one of the most important applications, as cracks provide critical information regarding structural condition and deterioration [1,2,3,4,5,6,7,8]. Depending on the type and location of a structure, crack characteristics such as width, orientation, and length must be examined [9,10,11]. In image-based UAV inspections, the interpretation of crack-related information is inherently influenced by the imaging resolution, typically expressed as the ground sampling distance (GSD) [1,11,12].

In general, a smaller GSD enables finer spatial representation of cracks, whereas a larger GSD limits the minimum resolvable crack thickness and increases uncertainty in crack interpretation [1,4,5,11,12,13]. At the same time, GSD is closely linked to flight configuration, including the distance between the camera and the target surface. This distance affects the image footprint, image quantity, and data-processing effort [14,15,16,17,18]. As a result, UAV-based crack inspection inevitably involves trade-offs between imaging resolution, operational efficiency, and measurement uncertainty. In practice, an excessive pursuit of the smallest possible GSD is often observed in UAV inspections, which can unintentionally increase data volume and processing burden without guaranteeing proportional gains in interpretability. Inspection planning, therefore, requires balanced consideration of GSD, flight distance, image quantity, and processing constraints rather than reliance on resolution alone.

Previous studies have discussed the relationship between flight distance and GSD based on camera imaging geometry and theoretical formulations. For example, Zhou et al. (2024) developed a UAV-based bridge inspection system emphasizing appropriate flight parameters for effective crack detection [1], while Shan et al. (2024) highlighted the importance of flight configuration in UAV-based pavement crack inspection [18]. Although these studies recognize the linkage between flight distance and imaging resolution, empirical investigations based on real UAV inspection flights remain relatively limited.

The relationship between GSD and crack interpretation has also been examined in prior work. Cho et al. (2018) showed that crack width estimation error is influenced by the number of pixels representing the crack, and proposed a probabilistic model to describe associated uncertainty [19]. Kye et al. (2021) further demonstrated that pixel-level representation, rather than GSD alone, plays a dominant role in crack width estimation accuracy, suggesting that resolution should be considered together with acquisition and imaging parameters [20]. These findings underscore that crack interpretation from imagery is fundamentally constrained by pixel-level representation, and thus GSD should be considered jointly with acquisition parameters rather than treated as an isolated factor.

In addition, the relationships among GSD, image quantity, and photogrammetric processing effort are complex. While smaller GSD values generally require more images to cover a given area, leading to increased processing load, larger GSD values may reduce image quantity and processing time [18,20,21]. However, explicit empirical analyses quantifying how these factors interact under practical UAV inspection conditions remain scarce.

Ultimately, these studies suggest that the selection of GSD and image acquisition parameters should be tailored to inspection objectives and practical constraints. In other words, the need for high resolution should be balanced against practical considerations such as processing capacity and time constraints [18,22,23]. To support such planning decisions, it is necessary to empirically examine the relationships among the key parameters affecting UAV-based crack inspection under realistic acquisition and processing conditions.

Unlike prior UAV crack inspection studies, which primarily focus on improving detection performance or reporting case-specific implementations, this study adopts a measurement-oriented perspective. It connects acquisition-side parameters (flight distance, GSD, image quantity, and processing effort) with interpretation-side uncertainty near the resolution limit. By combining empirical UAV flight evidence with a pixel-level uncertainty analysis that isolates resolution-driven instability. The results clarify why higher resolution does not necessarily translate into proportionate gains in interpretability. They also suggest how practical inspection planning can better balance efficiency and reliability.

Motivated by these gaps, this study presents an exploratory, measurement-oriented analysis of UAV-based crack inspection. The objective is not to propose a new crack detection or measurement algorithm, but to empirically examine how imaging resolution and operational parameters jointly influence inspection efficiency and pixel-level interpretation uncertainty. Specifically, the following research questions are addressed: (q1) the relationship between flight distance and average GSD; (q2) the relationship between average GSD and image quantity; (q3) the relationship between image quantity and image-processing effort; and (q4) the relationship between pixel-level crack thickness representation and segmentation-related uncertainty.

This paper contributes to UAV-based crack inspection research in three ways. (1) It provides empirical UAV flight evidence on how flight distance, GSD, image quantity/density, and photogrammetric processing effort interact under inspection-oriented acquisition constraints, highlighting that data volume and workload are not governed by GSD alone. (2) It introduces a complementary, dataset-based analysis that isolates resolution-driven interpretation uncertainty by quantifying how pixel-level crack representation in pixel units affects segmentation-related instability near the resolution limit. (3) It integrates the two perspectives to provide planning-level implications that balance operational efficiency and uncertainty-aware interpretability, rather than presenting a case-specific implementation or a new detection algorithm.

The remainder of this paper is organized as follows. Section 2 describes the materials and methods used in this study. Section 3 presents the results obtained from empirical UAV flight experiments and dataset-based image analysis. Section 4 discusses the implications of the observed relationships for UAV inspection planning, and Section 5 summarizes the main conclusions and limitations of the study.

## 2. Methods and Materials

### 2.1. Overview of Study Design

This study consists of two complementary analyses designed to examine UAV-based crack inspection from a measurement-oriented perspective. The first analysis is based on empirical UAV flight experiments conducted on a real civil structure. It examines how operational and imaging parameters, such as flight distance, ground sampling distance (GSD), image quantity, and photogrammetric processing effort, interact under practical inspection conditions. The second analysis is a dataset-based crack segmentation study intended to examine pixel-level uncertainty associated with crack thickness representation as image resolution approaches its practical limit. This segmentation analysis does not aim to validate crack measurement or segmentation performance, but rather to identify intrinsic limitations in image-based crack interpretation.

Based on this framework, the study adopts an exploratory and observational approach to investigate four research questions. Specifically, the relationships between flight distance and average GSD, average GSD and image quantity, and image quantity and image-processing effort are examined using data acquired from real UAV inspection flights. In addition, a separate dataset-based image analysis is conducted to examine the relationship between pixel-level crack thickness representation and segmentation-related uncertainty. All analyses are intended to identify descriptive trends under realistic inspection conditions, rather than to establish statistically validated causal relationships.

Rather than employing a factorial or fully controlled experimental design. Instead, it adopts an observational approach. One dominant parameter is varied across UAV flight missions, while other acquisition settings are held as constant as practically feasible. This approach reflects realistic inspection conditions and allows relationships between observed parameters and outcome variables to be explored without implying causal manipulation. For clarity, Table 1 summarizes the primary observed parameters and associated outcome variables analyzed for each research question.

### 2.2. Observational UAV Flight Experiments

#### 2.2.1. Test Site and Inspection Target

**Figure 1:**
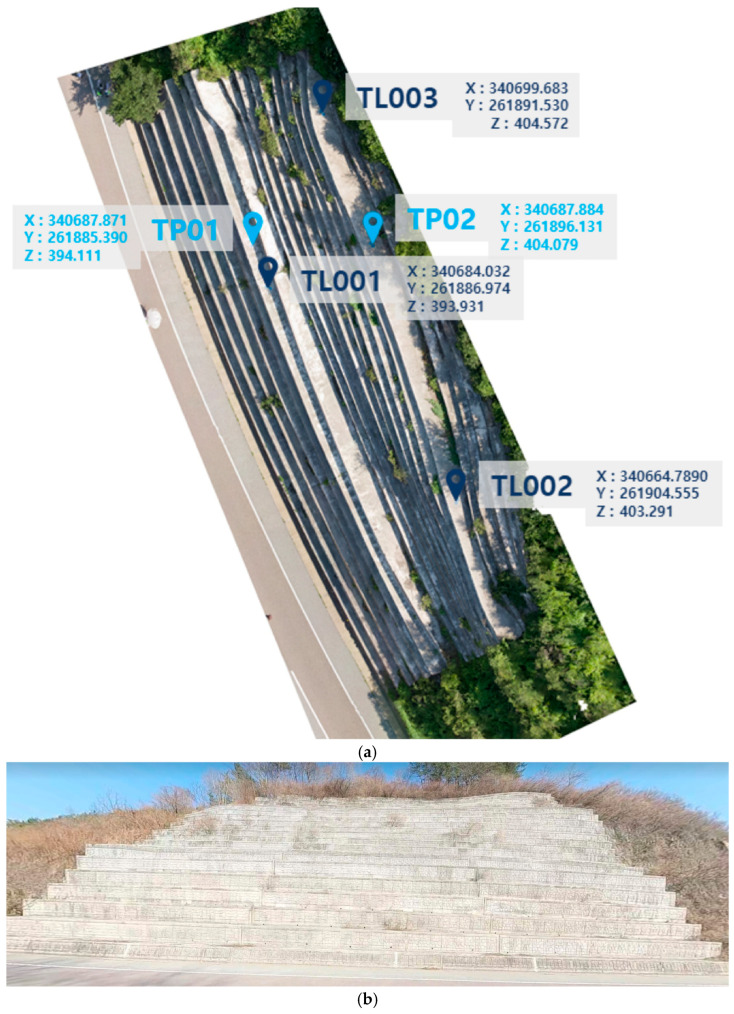
(**a**) Aerial image of the retaining wall and location of control points; (**b**) Road view of the retaining wall. Light blue label means Turning Point, dark blue label means Target Level.

The empirical UAV flight experiments were conducted at a reinforced concrete retaining wall located at an outdoor test site (Figure 1). The target structure consists of a planar wall surface with an approximate horizontal extent of 60 m and a vertical extent of approximately 6 m. The wall provides a sufficiently large and continuous surface for repeated UAV inspections conducted under different flight distances while maintaining comparable imaging conditions. The inspection target was selected to represent a typical civil infrastructure element for crack inspection, characterized by a relatively uniform surface texture and clearly visible surface cracks. The planar geometry of the wall allowed the effects of flight distance and imaging resolution to be examined without additional geometric complexity associated with curved or irregular structural surfaces. All UAV flights were conducted facing the wall surface with the camera oriented approximately normal to the target plane. This configuration was adopted to ensure consistent imaging geometry across flights and to facilitate comparison of image-based parameters derived from different flight distances.

#### 2.2.2. UAV Platform and Imaging Configuration

All flight experiments were conducted using a multirotor UAV equipped with an integrated RGB camera (Table 2). The camera system features a fixed focal length lens and a global shutter sensor, which were used consistently across all flight missions. Camera hardware characteristics, including focal length and sensor properties, were identical for all flights to ensure that variations in imaging parameters primarily resulted from changes in flight distance rather than camera configuration.

The camera was oriented approximately normal to the wall surface, with the optical axis maintained as perpendicular as practically feasible during flight. This imaging geometry was selected to reduce perspective distortion and to allow ground sampling distance (GSD) to be interpreted consistently as a function of camera-to-target distance.

Image acquisition was performed using automatic interval triggering during each flight mission. Exposure-related parameters were controlled by the onboard camera system to maintain stable image quality under outdoor lighting conditions. No user-defined camera calibration or lens distortion correction was applied during data acquisition. Although images were acquired from real UAV flights, the study did not explicitly quantify motion-induced blur or viewpoint perturbations; instead, a global-shutter camera and near-normal viewing were used to minimize such artifacts, and we treat their residual influence as a limitation/future work.

Camera intrinsic parameter optimization and distortion correction were performed during photogrammetric processing, as described in Section 2.2.4. This configuration ensured that the imaging system remained consistent across all missions, allowing observed differences in GSD, image quantity, and processing effort to be attributed primarily to variations in flight distance and mission geometry.

#### 2.2.3. Flight Planning and Data Acquisition

Because this study is based on empirical UAV inspection, flight planning, and data acquisition constitute a central component of the methodology. The overall inspection workflow followed commonly adopted UAV-based visual inspection practices reported in the literature. In this study, we focus on flight planning, data acquisition, and post-processing steps that directly influence imaging geometry and data characteristics.

Five UAV flight missions were conducted with nominal camera-to-target distances of approximately 6 m, 12 m, 20 m, 33 m, and 52 m, respectively. These distances are hereafter referred to as flight distances and represent the primary variable manipulated across missions. All flights employed the same UAV platform, camera system, and acquisition settings to minimize variability unrelated to flight distance.

Automated flight paths were generated using mission-planning software (ver 4.4.12), with image overlap treated as a controlled acquisition parameter. Rather than relying on nominal overlap percentages, image redundancy was evaluated post-flight using the Pix4D overlap map, which reports the number of overlapping images contributing to each pixel in the reconstructed orthomosaic. This approach was adopted because the inspection target is a vertical wall surface, for which conventional planar overlap definitions are not directly applicable.

Based on this design, flight plans were configured with the objective that pixels corresponding to the retaining wall surface would be covered by approximately five overlapping images. Post-flight overlap maps confirmed that the majority of wall pixels satisfied this criterion, indicating sufficient and consistent image redundancy for photogrammetric reconstruction. Overlap settings were held constant across missions. Therefore, variations in the number of calibrated images primarily reflect differences in imaging geometry associated with changes in flight distance and ground sampling distance. They are less attributable to the overlap design.

Image acquisition was performed automatically along the predefined flight paths. Flights were conducted under comparable environmental conditions as much as practically feasible, and missions were postponed when weather or illumination conditions could compromise image quality or operational safety.

#### 2.2.4. Image Selection and Definition of Image Quantity

Following UAV data acquisition, an image selection procedure was applied to define the input datasets for subsequent analysis in a consistent and reproducible manner. In this study, image quantity is defined as the number of images successfully registered during photogrammetric processing, rather than the total number of images initially captured during flight.

All captured images were imported into the photogrammetric processing software (4.4.12) and subjected to an initial image registration and bundle adjustment procedure. Images that were successfully registered and whose effective image footprints intersected the target retaining wall surface were retained for analysis. Images that failed registration or did not contribute to the reconstruction of the target wall surface were excluded. This selection criterion was applied uniformly across all flight missions and did not involve any manual or subjective filtering.

Image-level pre-processing steps, including lens distortion correction and radiometric normalization, were applied automatically as part of the photogrammetric processing workflow using identical software (4.4.12) settings for all datasets. No manual image enhancement, cropping, or editing was performed that could influence image content or crack appearance.

By defining image quantity based on successful image registration under controlled acquisition conditions, variations in image quantity across flight missions primarily reflect differences in imaging geometry associated with flight distance and ground sampling distance (GSD), rather than arbitrary image selection. This definition ensures that analyses related to image quantity (q2) and image-processing effort (q3) are based on objectively defined and reproducible datasets.

#### 2.2.5. Photogrammetric Processing and Processing Time Measurement

Photogrammetric reconstruction was performed using Pix4Dmapper (version 4.4.12) following an identical processing workflow for all datasets. The workflow consisted of image registration and bundle block adjustment, followed by orthomosaic generation. The same software version and processing options were applied to all flight datasets to ensure comparability of processing outcomes.

Image registration and bundle adjustment were carried out using the default Pix4D processing pipeline. Feature extraction and image matching were performed using consistent internal settings across all datasets, and camera parameters were optimized automatically during bundle adjustment. Automatic rematching was enabled to improve tie point robustness.

For representative datasets, approximately 86% of the input images were successfully registered. The mean reprojection error after bundle adjustment was approximately 0.21 pixels, indicating stable internal geometric consistency under the applied processing configuration. These metrics are reported to characterize relative reconstruction stability rather than to assess absolute reconstruction accuracy.

Ground control points (GCPs) were used to apply consistent georeferencing across datasets. Eight three-dimensional GCPs were employed, resulting in a mean root-mean-square (RMS) georeferencing error of approximately 0.012 m. Absolute positioning accuracy is not the primary focus of this study; rather, GCPs were used to ensure consistent reconstruction conditions across flight missions.

Image-processing time was recorded for the initial photogrammetric processing stage, excluding report generation and visualization steps. Processing times reported in this study should be interpreted as relative indicators obtained under a fixed software and hardware environment, rather than as absolute benchmarks. All processing was conducted on a single workstation equipped with an Intel^®^ Core™ i7-7800X CPU (3.50 GHz), 64 GB RAM, and an NVIDIA GeForce GTX 1060 GPU (3 GB), running Windows 10 Pro for Workstations (64-bit) (assembled in Seoul, Republic of Korea). Under these controlled conditions, differences in processing time primarily reflect variations in image quantity and reconstruction complexity.

### 2.3. Dataset-Based Crack Segmentation Analysis

#### 2.3.1. Purpose and Scope of the Segmentation Analysis

This subsection presents a dataset-based crack segmentation analysis conducted to examine pixel-level crack representation and segmentation-related uncertainty. This analysis is analytically separated from the empirical UAV flight experiments described in Section 2.2 and does not aim to validate physical crack width measurement or segmentation performance using UAV imagery.

The primary objective of this analysis is to investigate how crack scale, expressed in pixel units, influences segmentation behavior as image resolution approaches its practical limit. By focusing on pixel-level characteristics rather than physical crack dimensions, the analysis provides complementary insight into intrinsic limitations of image-based crack interpretation. This perspective is particularly relevant to UAV-based inspections, where ground sampling distance (GSD) determines the minimum number of pixels available to represent fine cracks.

Ideally, the relationship between GSD and crack width representation could be examined directly using UAV-acquired imagery with physical ground-truth measurements. However, such an analysis requires controlled imaging conditions and reliable reference measurements, which are beyond the scope of the present study. Instead, this subsection focuses on pixel-level crack representation as an intermediate and image-intrinsic quantity. By examining how segmentation outputs behave as crack thickness approaches the resolution limit, the analysis provides indirect insight into resolution-related uncertainty that is relevant when interpreting crack information from images.

#### 2.3.2. Crack Image Dataset

A publicly available crack image dataset was used for the segmentation analysis to ensure transparency and reproducibility. The use of an open dataset allows independent verification of the analysis procedure and avoids reliance on proprietary or inaccessible data sources.

This study adopts the crack dataset released by Lakshay Middha [25]. The dataset consists of 11,200 labeled crack images with a fixed spatial resolution of 448 × 448 pixels. For each RGB image, a corresponding binary mask is provided, in which crack regions are manually annotated at the pixel level. The dataset includes cracks with varying apparent thicknesses, continuity, and visual contrast, providing sufficient diversity for examining pixel-level crack representation across different crack appearances.

The dataset images were not acquired using UAV platforms and therefore differ from UAV imagery in terms of imaging geometry, perspective, and environmental conditions. Accordingly, the dataset is used exclusively to investigate pixel-level segmentation behavior and uncertainty, rather than to validate physical crack width measurement or UAV-based crack detection performance.

#### 2.3.3. Segmentation Model and Training Setup

A convolutional neural network based on a U-Net architecture [26] was employed to generate segmentation outputs for the crack images. The model follows a standard encoder–decoder structure with skip connections, which enables the preservation of spatial information and is commonly used for pixel-level segmentation of thin and elongated structures such as cracks.

The segmentation model was trained using the dataset described in Section 2.3.2, with binary crack masks used as ground-truth labels. The dataset was divided into training, validation, and test subsets to evaluate segmentation behavior under unseen data. Standard training procedures were applied, and no architectural modifications, advanced loss functions, or domain adaptation techniques were introduced.

It is emphasized that the purpose of using this model is not to demonstrate state-of-the-art segmentation performance or to benchmark crack detection accuracy. Rather, the model serves as a consistent tool for generating segmentation outputs that enable analysis of pixel-level crack representation and segmentation-related uncertainty. Accordingly, segmentation results are interpreted in terms of relative behavior and limitations, rather than absolute detection accuracy.

#### 2.3.4. Pixel-Level Crack Representation and Segmentation Uncertainty

To characterize the crack scale in image space, crack thickness was quantified in pixel units based on the annotated crack masks. For each connected crack region in the ground-truth mask, pixel-level representations were extracted to describe how cracks are expressed at the given image resolution. This representation reflects apparent crack thickness in the image domain and does not correspond to physical crack width in millimeters.

Segmentation-related uncertainty was evaluated by comparing predicted crack masks with the corresponding ground-truth masks at the pixel level. Discrepancies between predicted and annotated crack regions were interpreted as indicators of segmentation uncertainty rather than absolute detection errors. Differences in spatial extent, continuity, and thickness of segmented crack regions were examined to assess how segmentation behavior varies with pixel-level crack thickness.

Particular attention was given to thin cracks represented by only a small number of pixels. In such cases, minor pixel-level variations can result in disproportionately large relative differences in segmented crack thickness. This sensitivity highlights an intrinsic limitation of interpreting crack width directly from segmentation masks when crack representation approaches the resolution limit.

No attempt was made to convert pixel-based crack thickness into physical dimensions, and segmentation outputs are not interpreted as direct measurements of crack width. Instead, the analysis focuses on relative trends in segmentation behavior across different pixel-level crack scales to illustrate uncertainty characteristics inherent to pixel-based crack representation.

#### 2.3.5. Interpretation and Limitations

The dataset-based segmentation analysis presented in Section 2.3 is subject to several limitations. First, the crack dataset used in this analysis was not acquired using UAV platforms. As a result, the images do not reflect UAV-specific imaging effects such as motion-induced blur, perspective variation due to stand-off distance, or illumination changes commonly encountered during aerial inspections. This limits the direct transferability of the observed segmentation behavior to UAV-acquired imagery.

Second, segmentation uncertainty is inherently influenced by annotation quality and label ambiguity. Manually annotated crack masks may contain inconsistencies, particularly for thin cracks with diffuse boundaries where the distinction between crack and background pixels is not sharply defined. Consequently, discrepancies between predicted and annotated masks may partly reflect annotation uncertainty rather than model-related effects.

Third, the segmentation model employed in this study is not optimized for crack width estimation. Standard segmentation architectures trained with pixel-wise loss functions are known to be sensitive to thin and elongated structures, where small pixel-level deviations can produce relatively large variations in apparent crack thickness. Therefore, pixel-level thickness derived from segmentation masks should not be interpreted as a reliable estimator of physical crack width.

Despite these limitations, the purpose of this dataset-based analysis is not to replicate UAV imaging conditions or to validate crack width measurement performance. Instead, it focuses on pixel-level crack representation and the instability of segmentation-derived crack thickness as cracks approach the image resolution limit. Although the dataset images were not acquired using UAV platforms and therefore differ from UAV imagery in perspective, illumination, material appearance, and motion-induced artifacts, the uncertainty associated with thin-crack representation is an inherent characteristic of image-based interpretation and is not specific to a particular acquisition platform. Accordingly, the findings of this analysis are not intended to be transferred as quantitative crack-width estimates for UAV inspections; rather, they provide interpretative constraints on how segmentation outputs should be understood when cracks are represented by only a few pixels. In this sense, the results can inform UAV-based inspections as an uncertainty-aware interpretation guideline, rather than as a performance benchmark.

## 3. Results

This section presents the results obtained from the two complementary analyses conducted in this study. First, results from the empirical UAV flight experiments are reported to examine relationships among flight distance, ground sampling distance (GSD), image quantity, and image-processing effort (Section 3.1.1, Section 3.1.2 and Section 3.1.3). Second, results from the dataset-based segmentation analysis are presented to illustrate pixel-level crack representation and segmentation-related uncertainty (Section 3.2).

### 3.1. Empirical Results from UAV Flight Experiments (a1–a3)

#### 3.1.1. Relationship Between Flight Distance and Average GSD (a1)

**Figure 2:**
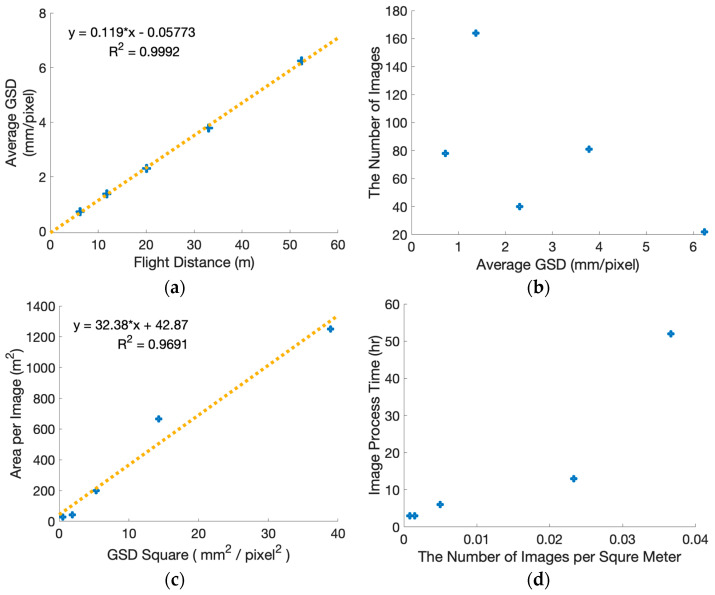
Relationship between parameters based on real flights: (**a**) flight distance vs. average GSD; (**b**) average GSD vs. the number of images; (**c**) GSD square vs. area per image; (**d**) the number of images per square meter vs. image processing time. Any fitted lines are shown only as visual guides to aid interpretation; no statistical inference is claimed due to the limited number of flight missions (*n* = 5).

Figure 2a shows the relationship between the flight distance and the average ground sampling distance (GSD) obtained from the empirical UAV flight experiments. As expected from basic camera imaging geometry, the average GSD increased with increasing flight distance. This trend was consistently observed across all five flight missions summarized in Table 3.

Within the investigated range of camera-to-target distances, the relationship between flight distance and average GSD follows the expected geometric proportionality. This behavior is consistent with the theoretical definition of GSD, which is proportional to the distance between the camera and the target surface when the camera intrinsic parameters are fixed. Small deviations from perfect linearity can be attributed to practical factors such as minor variations in camera orientation relative to the wall surface, spatial variation in image footprint across the target area, and averaging effects arising from multiple images contributing to the reconstructed products.

It is emphasized that this result does not constitute a novel finding. Rather, it serves as a verification that the UAV flight experiments were conducted in a physically consistent manner and that the acquired image datasets exhibit expected GSD behavior. Establishing this baseline is important for interpreting subsequent analyses involving GSD-dependent quantities, including image quantity and image-processing effort, which are discussed in Section 3.1.2 and Section 3.1.3. Therefore, given the exploratory nature and the limited number of missions (*n* = 5), we do not report regression statistics (e.g., *p*-values) and interpret the relationship only as a physically consistent trend.

#### 3.1.2. Relationship Between Average GSD and Image Quantity/Density Under Controlled Overlap (a2)

Figure 2b, together with the corresponding values in Table 3, presents the relationship between the average ground sampling distance (GSD) and the number of calibrated images obtained from the UAV flight experiments. As described in Section 2.2, image overlap conditions were intentionally kept constant across all flight missions, and image quantity was defined as the number of images successfully registered during photogrammetric processing.

Under controlled redundancy targets, average GSD did not show a monotonic relationship with the total number of calibrated images across the five flights. Specifically, the calibrated image count increased from 78 at 6 m to 164 at 12 m, dropped to 40 at 20 m, and then increased again at longer distances. This pattern is not interpreted as an experimental failure but as a practical characteristic of façade inspections, where the effective reconstructed extent can change with mission geometry. As stand-off distance increases, the camera footprint expands and non-target background (e.g., sky or ground) is more likely to be included in the photogrammetric block, which can alter registration outcomes and the final set of calibrated images. This is primarily because the reconstructed extent and effective surface coverage included in photogrammetric processing varied across missions. While larger GSD values were sometimes associated with fewer calibrated images, the overall distribution exhibits substantial variability. For example, the flight mission with an average GSD of 1.37 mm/pixel produced the largest number of calibrated images, whereas a mission with a moderate GSD of 2.30 mm/pixel resulted in a considerably smaller image count. This non-uniform behavior indicates that average GSD alone does not provide a reliable basis for predicting image quantity. In contrast, when image quantity is normalized by reconstructed surface area, the image density (images per area) exhibits a clearer inverse tendency with GSD, consistent with the expectation that coarser sampling reduces data density for a given overlap strategy.

The observed variability reflects the influence of factors beyond GSD, including acquisition geometry and flight-path configuration. Even under fixed overlap settings, differences in camera-to-target geometry, spatial extent of the reconstructed surface, and image footprint distribution across the wall surface contributed to variations in the number of calibrated images. Consequently, image quantity emerges from the combined effects of flight distance, scene coverage, and reconstruction geometry rather than from GSD alone. Therefore, our empirical results should be interpreted as showing that GSD more directly predicts data density, whereas total image count can be strongly influenced by mission geometry and reconstructed extent. Importantly, these results highlight that estimating data volume and processing workload in real inspections requires considering not only target redundancy and GSD but also scene framing and unavoidable background inclusion at larger stand-off distances.

#### 3.1.3. Relationship Between Image Quantity and Image-Processing Time (a3)

Figure 2d and Table 3 summarize the relationship between image quantity and photogrammetric image-processing time obtained from the empirical UAV flight experiments. Image-processing time refers to the duration required for the initial photogrammetric processing stage, including image registration and bundle adjustment, and was measured under a fixed software and hardware environment as described in Section 2.2.5.

Overall, image-processing time tended to increase with increasing image quantity. However, the relationship is not strictly linear. While datasets with higher image counts generally required longer processing times, reductions in processing time became less pronounced at lower image quantities. For example, a substantial decrease in image quantity did not result in a proportional reduction in processing time for the largest flight distances. This behavior suggests the presence of baseline computational overhead associated with image registration and bundle adjustment, which is incurred regardless of image count.

It is important to emphasize that image-processing time in photogrammetric workflows is influenced by multiple factors beyond image quantity, including hardware specifications, software version, internal processing settings, feature extraction density, and scene reconstruction complexity. In this study, these factors were held constant to the extent possible, and processing times are therefore interpreted only as relative indicators under controlled conditions, rather than as absolute or generalizable benchmarks.

Under these constraints, the observed results indicate that increases in image quantity are associated with increased processing effort, but the magnitude of this increase depends on the overall reconstruction context. Consequently, image quantity alone does not fully determine processing time, and reductions in image count do not necessarily yield proportional gains in computational efficiency.

### 3.2. Dataset-Based Segmentation Results (a4)

#### 3.2.1. Pixel-Level Segmentation Behavior Across Crack Thickness Scales

**Figure 3:**
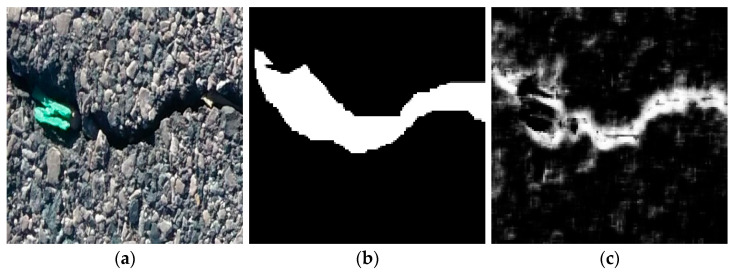
Representative examples from the dataset-based crack segmentation analysis: (**a**) input crack image; (**b**) human-annotated crack mask; and (**c**) segmentation output produced by the neural network.

Figure 3 presents representative examples from the dataset-based crack segmentation analysis, including an input crack image, the corresponding human-annotated mask, and the segmentation result produced by the neural network. These examples qualitatively illustrate how crack regions are represented at the pixel level and how segmentation outputs vary with local crack appearance and crack thickness.

**Figure 4:**
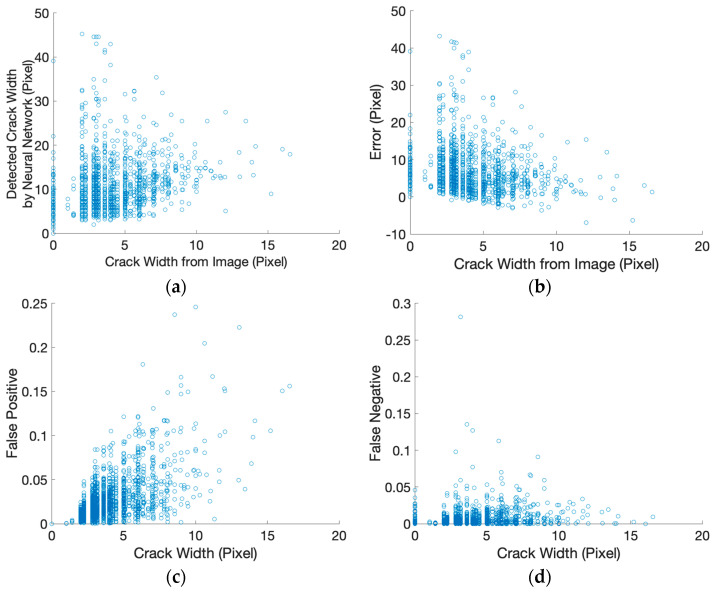
Pixel-level relationships between crack representation in annotated images and segmentation-related outputs: (**a**) crack width in the annotated image versus crack width derived from the segmentation mask; (**b**) pixel-level segmentation discrepancy; (**c**) false positive ratio; and (**d**) false negative ratio, shown as functions of crack thickness expressed in pixel units.

Figure 4 summarizes pixel-level relationships between crack thickness in the annotated images and segmentation-related outputs. In this analysis, crack thickness is expressed exclusively in pixel units derived from the ground-truth masks and does not correspond to physical crack width. As shown in Figure 4a, the crack thickness derived from the segmentation masks exhibits considerable dispersion relative to the annotated crack thickness. This dispersion becomes more pronounced for thinner cracks represented by only a few pixels.

Figure 4b illustrates the relationship between pixel-level crack thickness and segmentation discrepancy, expressed as absolute pixel-level error. Larger variability is observed for thinner cracks, indicating increased segmentation uncertainty as crack representation approaches the image resolution limit. Figure 4c,d present the distributions of false positive and false negative ratios as functions of crack thickness expressed in pixel units, further indicating that segmentation-related uncertainty is more prominent for thin cracks.

To quantitatively summarize these trends, crack thickness was grouped into three pixel-width categories (≤3 pixels, 4–7 pixels, and ≥8 pixels), and the segmentation discrepancy was evaluated using the absolute pixel-level error between annotated and segmented crack thickness (Table 4). Thin cracks (≤3 pixels) show the largest relative instability, with high dispersion in segmentation-derived thickness and elevated error variability near the pixel-resolution limit. The intermediate group (4–7 pixels) exhibits comparatively more stable behavior, while the thickest group (≥8 pixels) shows renewed variability consistent with increased boundary complexity for wider crack regions. In addition, standard mask-overlap metrics on the held-out test subset are reported to provide a quantitative reference for the segmentation outputs used in this uncertainty analysis (Table 5). These results quantitatively confirm that segmentation-derived crack thickness becomes increasingly unstable as crack representation approaches the pixel-resolution limit.

It should be noted that the absolute values of mIoU and Dice reported in Table 5 should not be interpreted as indicators of poor segmentation model performance. Instead, these low overlap scores primarily reflect resolution-limited instability when crack representation approaches the pixel threshold, where small boundary perturbations lead to large relative changes in mask overlap.

#### 3.2.2. Summary of Segmentation Uncertainty Characteristics

The dataset-based segmentation results reveal consistent patterns in how pixel-level crack representation influences segmentation behavior. Across the analyzed samples, segmentation outputs exhibited increasing variability as crack thickness expressed in pixel units decreased.

Although the dataset-based analysis in Section 3.2 is primarily intended to characterize resolution-driven uncertainty rather than to benchmark segmentation performance, we additionally report standard segmentation metrics to provide a quantitative reference. Thus, mIoU, Dice (F1), and pixel-wise Precision/Recall on the held-out test subset are reported as a quantitative reference. These metrics are reported as an auxiliary quality check and are not interpreted as validated crack-width measurement performance. As summarized in Table 5, overlap metrics (mIoU/Dice) and recall decrease markedly for the ≤3 px bin, indicating reduced stability near the resolution limit, whereas thicker bins show progressively higher and more consistent scores.

For cracks represented by multiple contiguous pixels, segmentation outputs tended to be spatially more continuous and less sensitive to local variations. In contrast, thin cracks near the image resolution limit showed greater sensitivity to local image contrast, annotation boundaries, and minor pixel-level variations. As a result, segmentation-related uncertainty was more pronounced for these thin crack structures, as reflected by higher dispersion and elevated false positive and false negative ratios.

Overall, thinner cracks exhibit greater dispersion and higher discrepancy variability, while thicker cracks show improved overlap metrics. These quantitative results characterize the uncertainty inherent in pixel-based crack representation and segmentation behavior. Accordingly, the results in Section 3.2 are interpreted as descriptive indicators of segmentation instability rather than as measures of crack width estimation capability.

## 4. Discussion

### 4.1. Interpretation of Empirical UAV Flight Results (a1–a3)

The empirical UAV flight experiments clarify how key imaging and operational parameters interact under practical inspection conditions. The relationship between flight distance and average GSD (a1) followed the expected geometric trend, confirming that the flight experiments were conducted in a physically consistent manner. Although this relationship is theoretically well established and often assumed in UAV inspection studies [19,22], explicit empirical verification using inspection-oriented UAV flight data remains limited. In this context, the present result serves to confirm that the acquired datasets exhibit physically consistent imaging behavior, thereby providing a reliable baseline for interpreting subsequent GSD-dependent analyses.

Under controlled overlap conditions, no clear monotonic relationship was observed between average GSD and image quantity (a2). This finding highlights that image quantity cannot be explained by GSD alone when overlap requirements are fixed. Instead, image count emerges from a combination of factors, including acquisition geometry, reconstructed surface extent, and flight-path configuration. In practical façade inspections, increasing stand-off distance expands the camera footprint and increases the likelihood of capturing non-target background regions (e.g., sky and ground) within the photogrammetric block. Such unavoidable scene inclusion can change the effective reconstructed extent and registration outcome, thereby influencing the total number of calibrated images even when redundancy targets on the wall are maintained. Accordingly, GSD is more directly informative for data density, whereas the total image count can be dominated by mission geometry and scene framing. This observation is particularly relevant for practical mission planning, where image quantity is often implicitly assumed to scale directly with resolution-related parameters, despite recent system-level UAV inspection frameworks that explicitly emphasize the influence of operational and mission-level constraints on image acquisition and processing effort [19].

The analysis of image quantity and photogrammetric processing time (a3) further demonstrates that processing effort increases with image density under a fixed software and hardware environment. However, the relationship is not strictly linear, and diminishing reductions in processing time were observed at lower image densities. This suggests the presence of baseline computational overhead associated with image calibration and bundle adjustment, regardless of image quantity.

Taken together, the results of a1–a3 emphasize that UAV-based crack inspection efficiency is governed by the interaction of multiple parameters rather than by any single variable. These findings support the need for integrated consideration of flight distance, GSD, overlap, and processing constraints during inspection planning. From a planning perspective, these findings suggest that mission design should explicitly manage scene framing and non-target inclusion (e.g., façade-parallel trajectories, controlled camera pointing, and ROI-aware processing) to improve the predictability of data volume and processing effort at larger stand-off distances.

### 4.2. Implications of Pixel-Level Segmentation Uncertainty (a4)

The dataset-based segmentation analysis (a4) provides complementary insight into the limitations of interpreting crack width from image-based segmentation outputs. By focusing on pixel-level crack representation rather than physical crack width, the analysis reveals that segmentation behavior becomes increasingly unstable as crack thickness approaches the image resolution limit. Although the segmentation analysis is conducted using a non-UAV dataset, it is intentionally designed to isolate pixel-level representation effects that are independent of the acquisition platform. These effects are directly relevant to UAV-based inspections because image resolution and pixel-level crack representation ultimately constrain the interpretability of segmentation outputs regardless of the imaging platform. Similar challenges related to thin crack representation and pixel-level ambiguity have been widely reported in recent UAV-based crack segmentation studies [5,13].

This study intentionally adopts a publicly available non-UAV dataset to separate pixel-level representation effects from UAV-specific imaging confounders (e.g., motion blur, viewpoint variation, and illumination changes). This design choice allows the analysis to focus on an intrinsic limitation of image-based interpretation—namely, the instability of segmentation-derived thickness when cracks approach the pixel-resolution limit—rather than conflating this effect with acquisition-specific artifacts. The resulting observations are therefore interpreted as a resolution-driven constraint that can inform how UAV-derived crack information should be read and used.

Thin cracks represented by only a few pixels exhibited greater dispersion in segmentation outputs and higher sensitivity to local image contrast and annotation boundaries. These characteristics indicate that pixel-level crack thickness derived from segmentation masks is inherently uncertain and should not be interpreted as a reliable estimator of physical crack width. This limitation is intrinsic to image-based representation and is not specific to a particular segmentation model.

Although the segmentation analysis was conducted using a non-UAV dataset, the observed uncertainty characteristics are relevant to UAV-based inspections insofar as they highlight fundamental constraints imposed by image resolution. When GSD increases or when cracks approach the minimum resolvable scale, segmentation-derived width estimates become increasingly sensitive to small pixel-level variations. This underscores the importance of cautious interpretation of crack width inferred from image segmentation, particularly for fine cracks near the detection limit.

For cracks approaching the resolution limit, improving pixel-level stability may require techniques beyond single-view segmentation. Recent studies have shown that super-resolution reconstruction can enhance crack detectability and downstream evaluation performance, particularly under limited pixel resolution conditions (e.g., SrcNet-based approaches and SR–segmentation pipelines) [27,28]. In addition, multi-view or multi-modal fusion frameworks have been explored to improve crack segmentation robustness by integrating complementary viewpoints or channels, sometimes with an SR step for resolution alignment prior to fusion [29]. While these methods are outside the scope of the present exploratory analysis, they provide actionable directions for future UAV inspections where cracks are near the GSD-imposed detectability limit [16].

### 4.3. Practical Implications for UAV-Based Crack Inspection Planning

The combined findings of this study provide practical implications for UAV-based crack inspection planning by clarifying (i) how acquisition parameters translate into operational workload and (ii) where pixel-level interpretation becomes intrinsically unstable. The empirical UAV results indicate that reducing flight distance decreases GSD and improves visual crack detectability, but it does not guarantee predictable changes in total image quantity because mission geometry and reconstructed extent can vary under real inspection conditions. Therefore, inspection planning should distinguish between data density (which tends to increase as GSD decreases under a given redundancy strategy) and total data volume, which can be strongly affected by scene framing and the inclusion of non-target backgrounds at larger stand-off distances.

The dataset-based segmentation analysis complements these operational observations by isolating a fundamental constraint: as crack thickness approaches the pixel-resolution limit, segmentation-derived crack thickness becomes increasingly variable and sensitive to minor pixel-level variations. Importantly, this uncertainty is not presented as UAV crack-width measurement performance; rather, it serves as a conceptual limitation that applies whenever crack interpretation relies on pixel-level representation. From a practical perspective, this implies that “excessive pursuit of high resolution” may not proportionally improve measurement interpretability once the workflow is dominated by segmentation uncertainty and boundary ambiguity for thin cracks.

Accordingly, the practical contribution of this study is not to claim validated physical crack-width measurement from UAV imagery, but to provide an integrated, measurement-oriented perspective on inspection planning: (1) empirical evidence of how flight distance, GSD, and mission geometry jointly affect data volume and processing effort in a realistic photogrammetric workflow; and (2) a resolution-limited interpretation constraint showing that pixel-based crack thickness inferred from segmentation masks should be treated as an approximate indicator, especially for fine cracks near the detection limit. These insights can support conservative planning choices by prioritizing a GSD that ensures cracks of interest are represented by a sufficient number of pixels, while also managing scene framing to avoid unnecessary data growth and processing overhead.

Finally, the findings motivate practical development directions for improving stability near the resolution limit, including multi-view fusion (to increase effective redundancy on the target plane), super-resolution or deblurring for UAV imagery, and width-estimation procedures that explicitly model thin-structure uncertainty (e.g., skeleton-based representations and distance-transform-based thickness estimation with calibration). Such developments can build upon the planning-level insights presented here to establish more robust inspection protocols.

### 4.4. Limitations and Scope of the Study

Several limitations of this study should be acknowledged. First, the empirical flight experiments were conducted at a single test site with a planar retaining wall, and the number of flight missions was limited. Flights were conducted under broadly similar illumination and weather; the effects of illumination change, shadows, moisture, and surface texture were not isolated. As a result, the observed relationships should be interpreted as exploratory rather than universally generalizable.

Especially, the empirical flights were conducted on a relatively planar concrete retaining wall, which provides a controlled inspection surface and reduces geometric complexity in photogrammetric reconstruction. Therefore, the observed relationships among flight distance, GSD, image density, and processing effort are most directly applicable to inspection tasks where the target surface is approximately planar or can be locally approximated as planar (e.g., retaining walls, large flat piers, or segments of bridge decks). For more complex structures such as bridges and tunnels, additional factors may affect the same relationships, including surface curvature, self-occlusion, varying viewing angles, non-uniform texture, and changes in effective camera-to-surface range across the scene. These factors can increase image redundancy requirements and influence calibration robustness and processing effort even under similar nominal GSD settings. Accordingly, the findings of this study should be interpreted as exploratory baseline evidence obtained under a controlled geometric scenario, while extension to curved or highly three-dimensional structures requires additional flight campaigns that explicitly account for curvature, occlusion, and viewpoint variation. Future work should validate the observed trends on representative bridge and tunnel geometries by controlling for curvature-driven range variation and by reporting reconstruction-quality indicators (e.g., calibrated image ratio, tie points, reprojection error).

Second, the dataset-based segmentation analysis was not performed on UAV-acquired imagery and does not account for UAV-specific imaging artifacts (e.g., motion-induced blur and viewpoint perturbations). While this analysis provides insight into resolution-driven pixel-level instability, further studies using UAV-acquired crack datasets with physical ground-truth measurements are required to validate segmentation stability under real inspection conditions. In particular, future empirical validation can be conducted via controlled UAV flight campaigns in which the same target is repeatedly inspected while systematically varying flight distance (GSD) and platform motion conditions (e.g., speed and viewing angle) to induce different levels of motion blur and perspective variation. Such a design would enable direct quantification of how GSD and UAV-specific artifacts jointly influence segmentation stability.

Third, processing time results are specific to the software version, settings, and hardware environment used in this study. Although relative trends were identified, absolute processing times may differ under different computational configurations.

Despite these limitations, the present study provides a structured empirical perspective on the trade-offs among flight distance, GSD, image quantity, processing effort, and segmentation uncertainty. By explicitly framing the analysis as exploratory, the results contribute to a clearer understanding of practical constraints relevant to UAV-based crack inspection planning.

## 5. Conclusions

This study explored the relationships among flight distance, ground sampling distance (GSD), image quantity, image processing time, and segmentation-related uncertainty in UAV-based crack inspection. An exploratory analytical framework combining empirical UAV flight experiments with a dataset-based crack segmentation analysis was adopted to examine resolution–efficiency trade-offs under practical inspection conditions.

The empirical flight experiments confirmed the expected geometric relationship between flight distance and average GSD, establishing a physically consistent basis for subsequent analyses. Under controlled redundancy targets, average GSD did not monotonically predict the total number of calibrated images across flights because the reconstructed extent and mission geometry varied; however, GSD showed a clearer inverse tendency with image density (images per reconstructed area). Accordingly, GSD is more informative for estimating data density than for predicting total image count in inspection-oriented missions.

The dataset-based segmentation analysis further demonstrated that pixel-level crack representation strongly influences segmentation behavior. As crack thickness approaches the image-resolution limit, segmentation outputs exhibit increased variability, highlighting intrinsic uncertainty in interpreting crack width directly from segmentation masks. These results indicate that segmentation-derived crack thickness should be regarded as an approximate indicator rather than a precise measurement of physical crack width, particularly for fine cracks.

Taken together, the findings suggest that effective UAV-based crack inspection requires balancing inspection efficiency and interpretability rather than indiscriminately maximizing image resolution. Selecting a GSD that ensures cracks of interest are represented by a sufficient number of pixels is more critical than pursuing minimal GSD values at the expense of increased operational and processing costs. At the same time, segmentation-based crack interpretation should be applied with caution near the resolution limit.

This study does not propose a validated quantitative design rule for UAV-based crack inspection. Instead, it provides preliminary empirical insights into how key imaging, operational, and interpretation-related parameters interact under realistic inspection constraints. Future work should include additional UAV flight experiments across diverse structural conditions, UAV-acquired crack datasets with physical ground-truth measurements, and improved crack width estimation methods to establish robust quantitative inspection guidelines.

## Figures and Tables

**Table 1:** Primary observed parameter and associated outcome variable for each research question.

Research Question	Primary Observed Parameter	Associated Outcome Variable
q1	Flight distance	Average GSD
q2	Average GSD	Image Quantity
q3	Image Quantity	Image Processing Time
q4	Pixel-level crack thickness in images	Segmentation-related discrepancy/uncertainty

**Table 2:** Specification of the camera and lens used in this study [24].

**Model**	Zenmuse X5S (Shenzhen, Guangdong, China)
**Lens**	DJI MFT 15 mm/1.7 ASPH
**Picture**	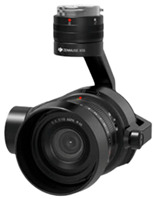
**Pixels**	5280 × 3956 (20.80 MP)
**Focal length**	15 mm
**FOV**	72 degrees

**Table 3:** Flight parameters acquired from empirical flights.

Flight Distance (m)	Average GSD (mm/pixel)	The Number of Calibrated Images	Reconstructed Image Coverage Area (m^2^)	Images Per Reconstructed Area (Images/m^2^)	Image Processing Time (hr)
6.18	0.72	78	2130	0.0366	52
11.8	1.37	164	7040	0.0233	13
20.1	2.30	40	7980	0.0050	6
33.0	3.78	81	54,900	0.0015	3
52.4	6.24	22	27,000	0.0008	3

**Table 4:** Summary statistics of segmentation-related discrepancy by pixel-level crack thickness bin.

Crack Thickness (Pixel)	Count	Mean	Standard Deviation	Variance	Median	IQR (Q1–Q3)
≤3 px	1147	5.38	5.13	26.31	4.00	2.24–6.20
4–7 px	962	5.00	5.24	27.42	3.14	1.83–6.39
≥8 px	151	5.44	4.48	20.03	4.47	2.47–6.95

**Table 5:** Quantitative segmentation metrics on the held-out test subset.

Crack Thickness (Pixel)	Count	mIoU (Crack)	Dice (F1)	Precision	Recall
Overall (test, macro)	2260	0.22	0.32	0.59	0.26
≤3 px	1147	0.14	0.21	0.43	0.17
4–7 px	962	0.29	0.41	0.74	0.34
≥8 px	151	0.40	0.53	0.81	0.47

## Data Availability

The raw UAV imagery and related data generated and/or analysed during the current study are not publicly available due to national critical infrastructure security restrictions, which prohibit external distribution. In addition, the raw and reconstructed datasets are very large (approximately 1 TB) and are archived on offline storage. Non-sensitive, aggregated results supporting the findings of this study are available from the corresponding author upon reasonable request, subject to approval by the relevant authority.

## Abbreviations

The following abbreviations are used in this manuscript:

CMOS	Complementary Metal-Oxide-Semiconductor
CNN	Convolution Neural Network
DSM	Digital Surface Model
GSD	Ground Sampling Distance
RMS	Root Mean Square
UAV	Unmanned Aerial Vehicle
